# Work-related health symptoms among compost facility workers: a cross-sectional study

**DOI:** 10.1186/0778-7367-70-13

**Published:** 2012-06-12

**Authors:** Ramona Hambach, Jos Droste, Guido François, Joost Weyler, Ulrik Van Soom, Antoon De Schryver, Jan Vanoeteren, Marc van Sprundel

**Affiliations:** 1 Department of Epidemiology and Social Medicine, Occupational and Environmental Medicine, Faculty of Medicine and Health Sciences, Campus Drie Eiken, University of Antwerp, Universiteitsplein 1, BE-2610, Antwerp, Belgium; 2 Occupational Health Service, Mensura, Antwerp, Belgium; 3 StatUA Statistics Center, University of Antwerp, Antwerp, Belgium

**Keywords:** Belgium, Compost, Industry, Occupational health, Organic dust, Workers

## Abstract

**Background:**

Industrial composting is a relatively new and expanding activity. Several studies indicate that compost workers are at risk to develop health symptoms. The aim of this study was to assess the prevalence of work-related health symptoms among compost workers compared with control subjects.

**Methods:**

A questionnaire was distributed among 62 workers (31 exposed and 31 non-exposed workers). Data were analyzed using simple and multiple logistic regression analyses.

**Results:**

Workers exposed to organic dust reported significantly more often respiratory, irritation (e.g., eyes, nose and throat), gastrointestinal**,** and skin symptoms than the non-exposed group. Moreover, all work-related symptoms were significantly more often reported by exposed than non-exposed workers. After adjustment for smoking status and age, the associations between exposure and respiratory, gastrointestinal, and skin symptoms remained statistically significant, in particular if these symptoms were work-related.

**Conclusions:**

This study confirms that workers at compost facilities are at risk to develop occupational health problems, most likely related to organic dust exposure.

## Background

Industrial composting is a relatively new and expanding activity. In Europe, this expansion is partially related to European Council Directive 1999/31/EC of 26 April 1999, which aims at reducing the amount of municipal solid waste going to landfill. The composting process can be defined as a controlled biological degradation of organic waste under conditions that are predominantly aerobic. This process results in a final product that can be applied for agricultural or horticultural purposes [1].

The compost industry in Flanders, Belgium, is a small sector wherein a limited number of people are employed. The sector comprises 25 green (park and garden waste) compost facilities (approximately 63 workers) and eight vegetable, fruit, and garden waste (VFG) compost facilities (approximately 72 workers) (personal communication: Wim Vanden Auweele, Vlaco, non-profit organization). The two types of waste are composted separately. VFG waste is processed in closed buildings (indoor composting) whereas green waste is composted outdoors.

During the composting process, microorganisms (such as bacteria and fungi), their components and metabolites, such as endotoxins, ß-1,3 glucans, and mycotoxins, and their spores can be aerosolised as organic dust [2-4]. Several authors report that compost workers are often exposed to very high levels of bioaerosols [4-6]. According to Wouters et al. (2006), the highest exposure concentrations of bioaerosols are found in those jobs in which waste is intensively handled indoors [4].

As reviewed by Domingo et al. (2008), several studies have investigated health effects of organic dust in compost workers [5]. These workers are at risk of developing respiratory, influenza-like symptoms, gastroenterological complaints, and irritation of eyes, nose, and skin [2,6,7]. Yet, the mechanisms that may induce these health effects are still unclear [6].

The aim of this study, carried out in Flanders, Belgium, was to estimate the occurrence of work-related health symptoms among VFG compost workers in three indoor facilities, compared with a non-exposed control population.

## Methods

The study design was cross-sectional. The Medical Ethics Committee of the University of Antwerp approved this study. A total of 62 male full-time workers participated. All participants gave their written informed consent.

Two Belgian External Occupational Health (OH) services (also called ‘external services for prevention and protection at work’) were approached through the professional network of the involved investigators and agreed to collaborate. Both are non-profit organisations, authorized by the Belgian labour legislation to provide occupational safety and health services to workers and employers [8].

Each OH service proposed affiliated VFG compost companies to be included for the study. Selection criteria were daily exposure to organic dust and voluntary participation. The exposure group comprised 31 male workers from three VFG indoor compost facilities located in Flanders, who worked almost exclusively in the compost hall, e.g., as a wheel loader driver, and/or as cleaning, and/or maintenance, and/or technical personnel and/or process operator. All of them carried out more than one of these tasks (job rotation). In addition, a non-exposed group with a similar socio-economic status (n = 31) was selected among warehouse workers at a pharmaceutical and surgical viscoelastics manufacturing plant, equally situated in Flanders. Selection criteria for the non-exposure group (one company) were no exposure to organic dust and chemical (including no exposure to diesel exhaust from forklifts) or biological agents, and voluntary participation. All selected companies and workers participated in the study. In addition, all facilities were located within a radius of 30 km from each other.

Data collection by questionnaire was incorporated into the annual medical examination of the personnel by the occupational health services between October and December 2005. All participants completed a validated questionnaire on compost-related health problems developed by the Institute for Risk Assessment Sciences (IRAS) from Utrecht University [9]. The questionnaire inquired, among other things, on symptoms suggestive of respiratory allergies and chronic respiratory symptoms such as cough, wheezing, dyspnoea, and phlegm production. In addition, a number of questions concerned irritation of eyes, nose, skin and gastroenterological symptoms. Finally, information was obtained on job history, working conditions and smoking habits of the participants. Individual symptoms were aggregated into several symptom groups when workers reported at least one of the following symptoms: respiratory symptoms (dry cough, phlegm, wheezing, chest tightness), irritation symptoms (runny eyes, blocking of nose, runny nose, sore throat, tickling nose or sneeze), gastroenterological symptoms (nausea, lack of appetite, pyrosis), and skin symptoms (skin rash). Symptoms were considered work-related if they were reported by the workers as being provoked or aggravated during their work (question ”Do you experience these symptoms during or shortly after work?”).

Data were analyzed using simple and multiple logistic regression analysis. Odds ratios (ORs) with 95% confidence intervals (CIs) not including the value 1 were considered to be statistically significant. Statistical analyses were performed using SPSS for Windows, v15.0 (SPSS, Inc, Chicago, Ill).

## Results

The distributions of the population characteristics are presented in Table 1. Exposed workers were younger and had a shorter seniority as compared with the non-exposed group. Furthermore, there were more current smokers among the exposed than among the non-exposed workers, whereas non-exposed participants had more frequently stopped smoking or more frequently never smoked than exposed participants. There were also differences in the prevalence of doctor-diagnosed (dd) allergy but not in dd asthma. Finally, more than half of the participating exposed workers did not wear a protective mask..

**Table 1:** Study population characteristics according to occupational organic dust exposure status, in numbers (percentages between brackets) (unless stated otherwise)

**Characteristics**	**No exposure**	**Exposure**
Number of employers	31	31
Male	31 (100)	31 (100)
Mean age, years (s.e.)^a^	43.8 (1.7)	37.1 (1.4)
Seniority, years (s.e.)^a^	10.9 (1.5)	7.0 (0.8)
Never	17 (54.8)	13 (41.9)
Ex	10 (32.3)	7 (22.6)
Current	4 (12.9)	11 (35.5)
Asthma (dd)^b^	2 (6.5)	2 (6.5)
Allergy (dd)^b^	4 (12.9)	1 (3.2)
P3 class mask	-	12 (38.7)
Gloves	-	31 (100)

^a^s.e.: standard error.

^b^dd: doctor-diagnosed

Table 2 shows the prevalence of reported individual symptoms and symptom groups by exposure group. All symptom groups and nearly all individual symptoms were more prevalent in the exposed groups. Exposed workers reported significantly more often respiratory, irritation, gastrointestinal, and skin symptoms than the workers in non-exposed group. After adjustment for age and smoking the association between exposure and respiratory, gastrointestinal, and skin symptoms remained statistically significant.

**Table 2:** Prevalence of symptoms (work-related or not) in workers according to organic dust exposure status, in numbers (percentages between brackets)

**Symptoms**	**No exposure (n = 31)**	**Exposure (n = 31)**	**Crude OR (95% CI)**	**Adjusted OR (95% CI)**^**a**^
**Respiratory**	**8 (25.8)**	**17 (54.8)**	**3.5 (1.2-10.2)**	**3.7 (1.1-12.0)**
Dry cough	5 (16.1)	10 (32.3)	2.5 (0.7-8.4)	3.2 (0.8-13.0)
Phlegm	5 (16.1)	9 (29.0)	2.1 (0.6-7.3)	2.0 (0.5-7.8)
Wheezing	1 (3.2)	2 (6.5)	2.1 (0.2-24.1)	2.2 (0.1-37.4)
Dyspnoea	2 (6.5)	3 (9.7)	1.6 (0.2-10.0)	2.5 (0.3-20.5)
Chest tightness	1 (3.2)	3 (9.7)	3.2 (0.3-32.7)	1.3 (0.1-17.3)
**Irritation**	**13 (41.9)**	**21 (67.7)**	**2.9 (1.0-8.2)**	**2.0 (0.6-6.4)**
Runny eyes	5 (16.1)	10 (32.3)	2.5 (0.7-8.4)	2.7 (0.7-10.0)
Blocking of nose	8 (25.8)	15 (48.4)	2.7 (0.9-7.9)	2.7 (0.8-9.1)
Runny nose	6 (19.4)	12 (38.7)	2.6 (0.8-8.3)	2.4 (0.7-8.3)
Sore throat	3 (9.7)	6 (19.4)	2.2 (0.5-9.9)	1.2 (0.2-6.4)
Tickling nose or sneezing	9 (29.0)	9 (29.0)	1.0 (0.3-3.0)	0.7 (0.2-2.3)
**Gastrointestinal**	**7 (22.6)**	**17 (54.8)**	**4.2 (1.4-12.5)**	**4.4 (1.2-15.5)**
Nausea	1 (3.2)	7 (22.6)	8.8 (1.0-76.1)	9.0 (0.9-85.3)
Pyrosis	6 (19.4)	12 (38.7)	2.6 (0.8-8.3)	2.5 (0.7-9.2)
Lack of appetite	0 (0.0)	3 (9.7)	-	-
**Skin**	**2 (6.5)**	**8 (25.8)**	**5.0 (1.0-26.1)**	**7.3 (1.0-52.0)**

^a^Multiple logistic regression analyses adjusted for smoking status and age.

The prevalence of work-related symptom groups by exposure status are summarized in Table 3. All work-related symptoms were significantly more often reported by exposed than non-exposed workers. After adjustment for smoking status and age, the statistically significant associations between exposure and work-related symptoms group persisted.

**Table 3:** Prevalence of work-related health symptom groups in workers by organic dust exposure categories, in absolute numbers (percentages between brackets)

**Symptoms**	**No exposure (n = 31)**	**Exposure (n = 31)**	**Crude OR (95% CI)**	**Adjusted OR (95% CI)**^**b**^
Respiratory	1 (3.3)	9 (29.0)	11.9 (1.4-100.7)	17.4 (1.7-178.4)
Irritation	4 (13.3)	11 (35.5)	3.6 (1.0-12.9)	4.7 (1.1-20.4)
Gastrointestinal	2 (6.7)	9 (29.0)	5.7 (1.1-29.3)	8.4 (1.3-52.9)
Skin	0 (0.0)	6 (20.0)	-	-
Any symptom	8 (26.7)	20 (64.5)	5.0 (1.7-14.9)	6.8 (1.8-25.3)

^a^Multiple logistic regression analyses adjusted for smoking status and age.

## Discussion

Our findings suggest that the compost workers participating in this study are more likely to report health symptoms than non-exposed subjects. Exposed subjects, in particular, reported significantly more often respiratory, gastrointestinal, and skin symptoms than those belonging to the non-exposed group. Moreover, all work-related symptoms were significantly more often reported by exposed than non-exposed workers.

As reviewed by Domingo et al. (2008) relatively few studies have investigated the health condition of compost workers [5]. This is the first study that investigated work-related health effects among compost workers in Flanders, Belgium. Composting of organic waste on a larger scale is a fairly new industrial activity in Flanders. Therefore, due to the limited number of workers active in this industry, the study population was small. This is a major limitation of this study since it could be the reason why some associations between exposure and health effects did not reach statistical significance. However, the strength of the associations suggests that the odds ratios found in our study are genuine. Following Santos et al. (2008), the OR is one of the most frequently used measures of association between a risk factor and an outcome (e.g. health effect) in epidemiology [9]. The risk ratio (RR) and prevalence ration (PR) are important measures to quantify the strength of an association between a risk factor and a health effect [9]. Thompson et al. (1998), stressed that the OR overestimates the RR or PR when the health effect is common (i.e., prevalence higher than 10%) [10]. As argued by Santos et al., the major draw-back of using OR when an outcome is common, is related to its misinterpretation as PR [9]. Furthermore, there are some other limitations of the present study. We cannot be certain that no selection bias ‘healthy worker effect (HWE)’ was introduced. The HWE refers to the phenomenon that workers must be relatively healthy in order to be employable in a workforce. As stated by Li et al. (1999), morbidity and mortality rates within the workforce are usually lower than in the general population. As a result, increases in both morbitity and mortality due to occupational exposure might be wholly or partially masked [11]. Another possible limitation of this study is the occurrence of reporting or recall bias. Citing Pearse and Checkoway (1988), ‘recall bias may occur because a patient with a chronic disease may ponder the possible causes of their disease, and therefore they may be more likely to recall some past exposures than healthy controls’ [12]. In addition, the cross-sectional design gives no information on the temporal sequence between exposure and outcome.

Several studies illustrate that exposure to organic dust in compost workers is significantly associated with a higher frequency of health symptoms and diseases [2,6,7]. For example, a cross-sectional study by Bünger et al. (2000) in 58 VFG compost workers and 40 control subjects described a significantly higher prevalence of skin diseases and respiratory symptoms among compost workers than in the reference population [2]. These results correspond well with our results. Unlike Bünger and colleagues, however, we found a significantly higher prevalence of work-related gastroenterological symptoms among exposed subjects. Gastroenterological symptoms among compost workers were also reported by other authors [3,7]. Furthermore, we found significantly more irritation symptoms of the eyes and upper airways in the exposed than in the non-exposed group, which is in line with results of Bünger et al. (2007) [6]. As far as we are aware of, only one longitudinal study on health problems in the compost industry has recently been published [6]. This five-year follow-up study showed that the number of compost workers with chronic bronchitis doubled during the observation period. Some authors reported a healthy-worker effect in subjects occupationally exposed to bioaerosols, suggesting that health risks may even be underestimated [2,13].

The mechanisms that may induce these health effects are still unclear [5,6]. According to Jaakkola et al. (2002), several possible mechanisms have been put forward such as IgE-mediated hypersensitivity reactions, toxic reactions due to mycotoxins and irritative reactions due to volatile organic compounds (MVOC) emitted by microorganisms [14]. The authors stress that it is probable that different microorganisms have their influence by different mechanisms. Wouters and colleagues (2002) underline that non-allergic inflammatory reactions may be important, especially due to dust containing endotoxins and β (1–3)-glucans, two known proinflammatory cell wall components of gram-negative bacteria and most fungi [15].

The most common fungi abundantly present in compost piles are *Aspergillus* spp., *Penicillium* spp., *Cladosporium* spp., and *Alternaria* spp. Some of these fungi (e.g., *Aspergillus* spp. and *Penicillium* spp.) can produce mycotoxins, which are harmful to human health [3]. Furthermore, case reports have shown the occurrence of hypersensitivity pneumonitis, allergic bronchopulmonary aspergillosis, and asthma in compost workers exposed to high concentrations of organic dust [16,17]. Moulds and thermophilic bacteria are well-known sources of allergens that may play a role in the development of hypersensitivity pneumonitis [18]. However, as cited by Wouters et al. (2002), ‘allergic diseases are rarely reported in surveys and are unlikely to explain the occurrence of most respiratory symptoms’ [15]. A relationship between endotoxin exposure and fever, respiratory problems and gastroenterological problems is described in several studies [19,20].

Tolvanen et al. (2005) concluded that compost workers were working in poor hygienic conditions [3]. Therefore the authors recommend that workers should wear personal protective equipment (e.g., gloves and a respiratory mask class P3). In addition, as advised by a Canadian study, workers should not work too long in the composting hall moreover the importance of personal hygiene should be emphasized [21]. The results of our study, in which less than half of the exposed workers used a respiratory mask, underline these recommendations.

## Conclusions

Our results demonstrate that workers at compost facilities have an increased risk of developing health problems, most likely related to occupational exposure to organic dust. The findings underline the need for an accurate and continuing evaluation of organic dust exposure and for the development and application of control strategies in compost facilities.

## Competing interests

The authors declared that they have no competing interest.

## Authors’ contributions

Each author has actively contributed to developing both the concept and design of the study. RH, JD, GF and MvS were the main contributors in writing the manuscript. All authors have read and approved the final version submitted.
